# Process Evaluation of Individual Placement and Support and Participatory Workplace Intervention to Increase the Sustainable Work Participation of People with Work Disabilities

**DOI:** 10.1007/s10926-024-10214-x

**Published:** 2024-06-25

**Authors:** E. Oude Geerdink, M. A. Huysmans, H. van Kempen, J. M. Maarleveld, J. van Weeghel, J. R. Anema

**Affiliations:** 1 Department of Public and Occupational Health, Amsterdam Public Health Research Institute, Amsterdam UMC - Location VU Medical Center, Vrije Universiteit Amsterdam, Van Der Boechorstraat 7, Postbus 7057, 1081 BT Amsterdam, The Netherlands; 2 Research and Statistics, City of Amsterdam, Amsterdam, The Netherlands; 3 Tranzo, Tilburg School of Social and Behavioral Sciences, Tilburg University, Tilburg, The Netherlands

**Keywords:** Labor market participation, Occupational health, Supported employment, Vocational rehabilitation, Social welfare

## Abstract

**Purpose:**

This study is a process evaluation of the use of Individual Placement and Support (IPS) and Participatory Workplace Intervention (PWI) to increase the work participation of people with work disabilities. We ran the evaluation alongside a randomized controlled trial (RCT), to investigate whether and to what extent IPS and PWI were executed according to protocol.

**Methods:**

The study population consisted of clients with work disabilities, and their job coaches who were employed by the municipality of a large city in the Netherlands. Data were collected between September 2019 and November 2022 using registration forms, accompanied by researchers’ notes and logbooks.

**Results:**

For IPS the dose delivered was reasonable and the IPS fidelity measurement score was fair. The job search focused on paid work for almost all clients and was based on their wishes as indicated in the protocol, but integration of employment services with (health) care was often lacking. A minority of the clients who were assigned to PWI received the intervention, often because the client did not start work within the follow-up period and a workplace was a requirement to apply the intervention.

**Conclusion:**

The results of this study show that IPS was executed reasonably and with a fair fidelity, which indicated implementation was sufficient to find an effect on work participation in the RCT. PWI was barely realized in practice and no conclusions regarding the fidelity could be drawn. We therefore conclude that we cannot expect PWI to have any effect on work participation in the RCT.

**Supplementary Information:**

The online version contains supplementary material available at 10.1007/s10926-024-10214-x.

## Introduction

The importance of paid work has been widely acknowledged. Employment has financial benefits and can increase health and wellbeing, whereas unemployment can negatively impact both spheres [1–3]. People with work disabilities, i.e., people who face physical, psychological, and/or social limitations that hinder them in finding and keeping a job, are supported with a variety of schemes that are put into place to improve their position in the labor market. However, despite widespread efforts, work participation among people with work disabilities remains low when compared to work participation among people without work disabilities [4, 5]. This illustrates the need for more effective interventions.

In the Netherlands the ‘Participation Act’ was put into place in 2015, to ensure that people who are not able to find and keep a paid job by themselves receive a minimum income in the form of welfare benefits, while also receiving support to find and keep a job. The provision of welfare benefits and support are the responsibility of local governments (i.e., municipalities), who have a certain degree of freedom with regards to how they arrange this support. This study took place in a large municipality in the Netherlands, where when citizens apply for welfare benefits a case manager evaluates their capacity to work. When a work disability is present or suspected, the client is referred to the Work Disabilities team. The client’s ‘readiness to work’ is then evaluated (i.e., whether they have sufficient basic employee skills to find and keep a paid job). For example, people who have had a paid or unpaid job or who were enrolled in an education program in the past 6 months are usually considered ‘ready to work.’ Clients who are considered ready then start a trajectory in which they are supported by a job coach to find and keep a paid job. Clients who are considered ‘not yet ready to work’ receive support based on the ‘first train, then place’ principle: they first receive support to become ready for work, mainly consisting of pre-vocational training and internships, and after that they also receive support from a job coach to find and keep a paid job.

To improve the success of these welfare-to-work services, we introduced two existing interventions in this setting: Individual Placement and Support (IPS) and Participatory Workplace Intervention (PWI) [6]. IPS has been shown to be effective at increasing competitive employment among people with severe mental illness, and recent studies have suggested that IPS might be effective for other populations as well (such as people with anxiety or musculoskeletal disorders) [7, 8]. PWI has been proven effective in decreasing time to return to work, especially among people with musculoskeletal disorders, but also for sick-listed employees with distress, who intended to return to work despite symptoms [9–11]. IPS and PWI were applied in the municipal setting, and we studied their effectiveness in improving work participation for people with work disabilities using a randomized controlled trial (RCT).

However, effectiveness trials alone are not enough to fully understand results and make recommendations for practice; when complex interventions (i.e., interventions that contain multiple components) such as IPS and PWI are studied, it is important that process evaluations are performed to gain insight into whether the interventions are executed as intended [12–14]. A process evaluation addresses the extent to which an intervention was delivered and how it was delivered and can also describe how the contextual factors of the study setting affected the implementation and study outcomes [15–17].

This paper reports on a process evaluation of IPS and PWI as complex interventions to increase work participation among people with work disabilities. The main research question was whether and to what extent IPS and PWI were executed according to protocol at a large municipality in the Netherlands.

## Methods

This process evaluation was performed alongside a 2 × 2 factorial RCT in which the effectiveness of IPS and PWI for people with work disabilities was examined [6]. For this trial, 118 people were randomized to receive either IPS or no-IPS (1:1 ratio) or PWI or no-PWI (1:1 ratio). This resulted in 4 groups: IPS, PWI, IPS + PWI, and Service as Usual (SAU). The follow-up of the trial was 18 months. The project took place in a large municipality in the Netherlands at the department for Work and Participation, where people with work disabilities are provided with welfare benefits and receive support to find paid employment or participate in society in another way that fits their capabilities. The Medical Ethics Committee of the Vrije Universiteit Medical Centre approved the study protocol and declared that it was not subject to the Medical Research Involving Human Subjects Act (2018.462). All participants gave written or oral informed consent before participation in the trial. In the Netherlands, adolescents of 16 years and older are allowed to give informed consent without involving parents or legal guardians. However, when clients felt more comfortable with a parent or other family member present, these were also invited to the inclusion appointment. More information about the RCT can be found in the pre-registration in International Clinical Trial Registry Platform (NL9771), and elsewhere [6]. Based on the framework of Linnan and Steckler, this process evaluation described the recruitment, reach, dose delivered, and fidelity of IPS and PWI [16]. Data collection took place between September 2019 and November 2022, using general and study-specific registration forms from job coaches, information from logbooks, and researchers’ notes.

### Study Population

#### Clients

Clients were approached to participate in the RCT when they (i) were recently referred to the Work Disabilities team, (ii) were willing to obtain competitive work, (iii) had a (suspected) work disability, and (iv) were 16 years or older. Clients were considered ‘recently’ referred to the Work Disabilities team when they were referred to the team but their trajectory had not started yet. In most cases they were contacted within 2 working days after their referral, but in some cases it took a couple of weeks before they could be contacted, due to administrative procedures. A work disability was defined as ‘not being able to provide your own income due to long-term (i.e., not expected to be solved within a year) physical, psychological, and/or social problems. Exclusion criteria were (i) being unable to give informed consent due to cognitive or language barriers or (ii) already having paid employment for 12 h or more per week.

#### Professionals

All job coaches who were working at the department where the RCT was conducted were invited to participate in the study. These job coaches all had experience with providing coaching to clients with work disabilities, aiming to find and keep paid employment. Job coaches who agreed to participate and who were assigned to the IPS, PWI, or IPS + PWI group were expected to participate in a training to become capable of providing the intervention. There were no exclusion criteria, however, job coaches were asked not to participate if they were planning on finding a different job themselves within a year.

### Interventions

#### Individual Placement and Support (IPS)

The main aim of IPS was to support people in obtaining and keeping a regular paid job based on the wishes of the clients, according to the so-called ‘first place, then train’ principle. This means that the job search started immediately, regardless of whether clients were considered ‘ready for work’ at intake. If necessary, additional training could then take place at the workplace. Because IPS was originally developed for healthcare settings and the coaching of people with severe mental illness, some adaptations had to be made in order to apply IPS in a municipal setting for clients with work disabilities (not only due to mental illness). Appendix 1 (supplementary file 1) shows an overview of the eight principles of IPS [7] and the adaptations that were made.

Job coaches who participated in the IPS group received an adapted IPS training that encompassed two days of theory and was supplemented with supervision meetings. The training was provided by a certified IPS trainer. All certified job coaches could participate in the IPS training. Since all participating coaches had ample experience with benefits counseling and job development, limited attention was paid to these topics. Instead, given that the job coaches in the study were not part of a healthcare treatment team, extra attention was paid to how to integrating job coaching and (mental) healthcare and to making contact with family and/or other (in)formal caretakers.

#### Participatory Workplace Intervention (PWI)

The objective of PWI was to increase sustainable employment by identifying and solving obstacles to work continuation at an early stage of employment. PWI is a conversation method led by a process leader that follows a stepwise approach. The goal is to have the worker and their supervisor or employer reach consensus on the most important obstacles for work functioning and on which actions to undertake to overcome these obstacles. An overview of the steps of PWI can be found in Appendix 2 (supplementary file 2).

PWI process leaders received a 4-h training before the start of the trial and an additional ‘refresher training’ 12 months after the start of recruitment of clients for the trial. In this training, professionals were provided with theory on PWI and on their role as process leaders, which was to remain impartial, help supervisors and employees find understanding for each other’s perspectives, and make sure both the supervisor and employee take responsibility for the success of PWI. Professionals also practiced the different steps of the intervention in the training, using the intervention materials consisting of conversation guides and forms that had to be filled in.

#### IPS + PWI

The RCT that was performed used a factorial design; in addition to the effectiveness of IPS and PWI, we also investigated whether a combination of both interventions increased impact. Therefore, approximately 25% of participants received IPS and PWI. For this process evaluation, we investigated the interventions separately.

### Process Measures

Below is a description of how the process measures in this study were defined and how they are reported in the results section of this paper.

#### Recruitment

Recruitment was defined as the procedures used to recruit job coaches and clients, and includes a description and the flow of clients in the process in a diagram.

#### Reach

Reach on the level of clients was defined as the proportion of the target population that participated, (i.e., the clients who participated in the study in the numerator and all eligible clients who were contacted by the researchers in the denominator). The flow diagram also reports the number of participants who were excluded and for what reasons, and the number of clients who refused to participate.

#### Dose Delivered

Dose delivered was defined as the extent to which the interventions were carried out by reporting the number of clients for whom each protocol step was completed. While PWI is a stepwise process, IPS is a continuous process in which there are no separate steps that need to be completed in a specific order. However, we distinguished four phases with multiple steps per phase to gain insight and report on the extent to which the protocol was followed.

We also determined intensity and duration of the interventions as part of the dose delivered. Information on duration and intensity was available for the first 12-months after inclusion for each client. Intensity was defined as the average time in minutes per week that job coaches spent per client. This included all coaching-related time investment, also including e.g., contact with healthcare providers. The total number of minutes was divided by the number of weeks that any time investment was reported for each client. Duration was reported as the average number of weeks between the first and last time that job coaches reported any time investment.

#### Fidelity

An IPS fidelity review was performed in March 2022 by two independent reviewers using the regular IPS fidelity scale [18]. Due to the unique setting of this study, some components of the fidelity review process could not take place (e.g., an interview with healthcare providers). To make sure the reviewers could obtain sufficient information, alternatives were offered if possible. For example, interviews with other important partners such as client coordinators and job hunters within the organization were organized. The fidelity review resulted in a report that included a general summary and detailed scoring information, which was made available to the researchers. In contrast to the other process measures in this study, the IPS fidelity review report provided information on the system level instead of on the individual client level.

For PWI, no standardized fidelity measurement instrument exists. Instead, we examined the content of the intervention materials qualitatively to assess the quality of the approach. In line with previous studies on participatory workplace interventions, we examined whether it was clear how obstacles were related to functioning at work, whether the solutions were related to these obstacles, and whether the action plan was clear and concise (i.e., whether it included who was responsible for the actions, when they were going to take place, and how they were going to be executed) [19, 20].

### Data Collection

Information on recruitment and reach was extracted from the databases that were used during the inclusion phase of the RCT. To determine the dose delivered, participating job coaches received registration forms in which they were asked to indicate which elements of the intervention or interventions had been carried out for each client they had coached during the study. The dose delivered was determined in November and December 2022 for all clients. At that point, all clients had participated in the trail for at least 1 year. During the trial, job coaches were asked to inform the researcher of the amount of time they invested in coaching every week. Information for the fidelity measurement of IPS was obtained by the independent reviewers.

### Data Analysis

Descriptive statistics were used to analyze the data, using SPSS (IBM Corp. Released 2021. IBM SPSS Statistics for Windows, Version 28.0. Armonk, NY: IBM Corp) and Microsoft Excel (Microsoft Corporation (2018)).

## Results

### Recruitment

#### Professionals

In April 2019, researchers joined team meetings at the municipality to invite job coaches to participate. We aimed to include at least three job coaches for each trial arm (IPS, PWI, IPS + PWI, and service as usual (SAU)). Only job coaches who were officially registered as a job coach could participate in the IPS-training, because then the IPS trainers were sure that they had sufficient experience with job coaching and sufficient knowledge on available schemes, subsidies, and laws and regulations related to vocational reintegration. We therefore aimed to only include job coaches who were officially registered. However, we were unable to recruit a sufficient number of registered job coaches who were willing to participate. In addition to this, some job coaches indicated they only wanted to participate if they would be assigned to a certain group. Therefore, we had to also include job coaches who were not registered, and we could not randomize on the level of job coaches. In this way we were able to include a sufficient number of job coaches, who were allocated to a group based on whether they were registered and on their preference. Job coaches who were not officially registered could be allocated to either PWI or SAU, and job coaches who were officially registered could be allocated to IPS, PWI, IPS + PWI, or SAU. All professionals who participated had at least 2 years of experience with coaching of clients with a work disability.

#### Clients

**Fig. 1:**
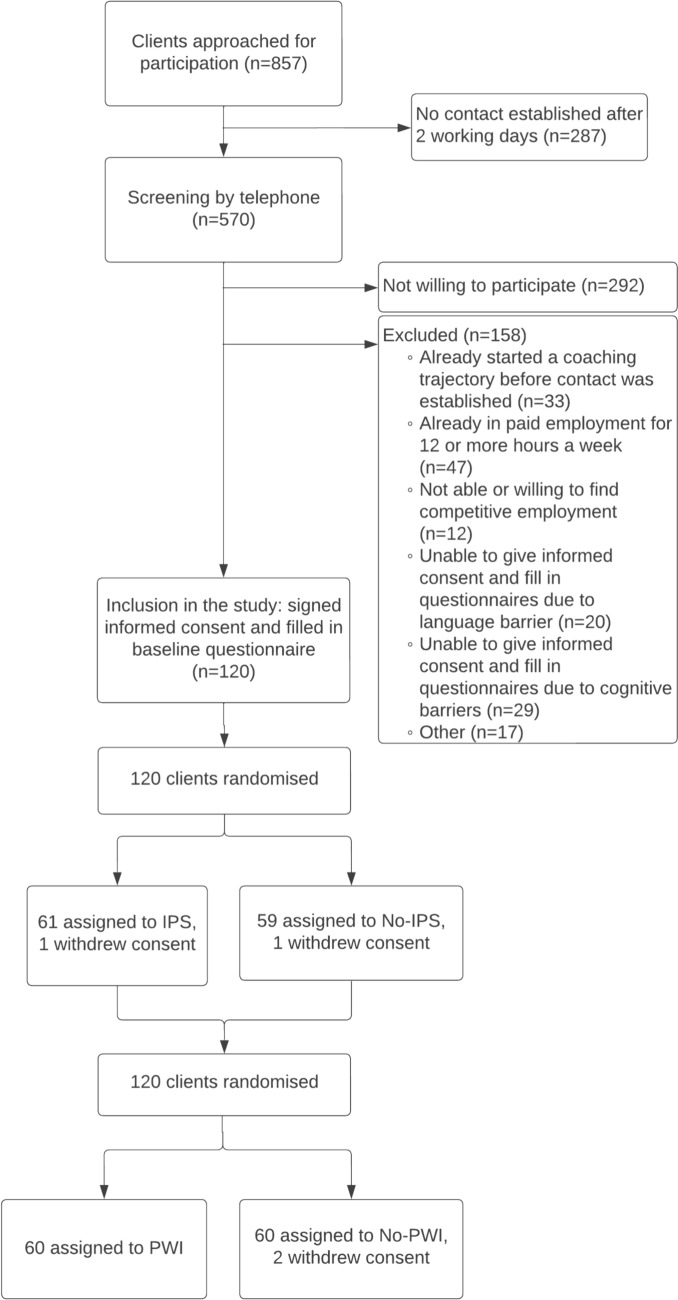
Recruitment of participants

An overview of the recruitment procedures that were used to include clients can be found in Box 1. We aimed to include 120 clients in the study. Recruitment of clients started in November 2019 and was finished in October 2021. In March 2020, recruitment was paused because of restrictions due to COVID-19. When everything was set up to recruit remotely (after approximately 6 weeks) inclusion was continued. Figure 1 shows the flow of clients in the study.

**Box 1:** Detailed description of recruitment of clients.

**Step 1—Screening of new applications** - Daily check whether new clients were admitted to the department by researcher/research assistant - Eligibility of new admissions was checked, based on information available in registration systems of the municipality **Step 2—First contact with researchers by phone: eligibility screening** - Text messages were sent to eligible clients, informing them that researchers would contact and invite them to participate in a scientific study - Eligible clients were informed about the study by phone; if they were interested, additional eligibility checks were performed - Clients who indicated interest in participation were provided with information about the study (consisting of a flyer and information letter) by a text message or email
**Step 3—Second contact with researchers by phone: decision on participation** - Clients who indicated interest were called back after one workday - When clients indicated they wanted to participate, an appointment was made for their inclusion in the trial **Step 4—Inclusion** - An appointment took place between the participant and researcher/research assistant, either face- to-face or digitally (using video calls or by telephone) - After going over the information letter together, an informed consent form was signed if the client was still willing to participate in the study - The job coach was informed that a new client had been added to their caseload

### Reach

#### Professionals

Fifteen job coaches volunteered to participate in the study. Most of them were trained in the intervention they preferred, but some were not assigned to their first choice in order to have enough job coaches for each group. Eight job coaches were trained in IPS and eight in PWI. Three job coaches stopped participation during the study and were replaced, or their caseload was divided among the remaining job coaches of that trial arm.

#### Clients

Figure 1 shows that 857 clients were approached, contact was established with 412 eligible clients, and 120 of these agreed to participate in the study. This resulted in a reach of 29%. Of these 120 clients, 61 were assigned to IPS (30 IPS and 31 IPS + PWI) and 60 were assigned to PWI (29 PWI and 31 IPS + PWI). Five clients turned out to be ineligible for coaching from the Work Disabilities team after inclusion, because they should have been admitted to a different department or organization according to regulations (2 in the PWI and 3 in the IPS group). Two clients withdrew their consent. These clients are not considered in the rest of our analyses.

### Dose Delivered

#### IPS

Table 1 shows the dose delivered for IPS. While job coaches assessed the network for most clients (72%), for less than half (46%) the most important members in these networks were contacted. When contact was established, it was most often with healthcare providers or other caregivers (46%) or with a partner or a family member (31%). For the remaining cases, it was unclear who was contacted. In most cases, job searches focused on finding competitive employment or regular education, based on the wishes of the client. Most clients (72%) started competitive employment at some point during the trajectory, but 34% did not receive continued coaching during their employment. Often this discontinuation was because the client said they no longer needed coaching, but sometimes it was due to practical reasons (e.g., COVID restrictions). Some clients performed voluntary work during the trajectory, either because they had already been volunteering and were at the same time looking for a regular paid job, or because they decided they wanted to start voluntary work at some point during the trajectory.

**Table 1:** Dose delivered—Individual Placement and Support

Phase	Steps	Number (%) of clients for whom this was executed (*N* = 57)
Intake	Assess network of the client: healthcare specialists, professional and non-professional caregivers, important family members/friends/partners	41 (72%)
	Contact important members of the network	26 (46%)
	Fill in intake & action form	48 (84%)
Assessment (continuous process)	Update assessment form continuously with new information throughout the trajectory	43 (75%)
Acquisition and placement	Search for competitive employment	55 (96%)
	Search based on wishes of the client	55 (96%)
	Client started competitive employment	41 (72%), of which 15 (37%) clients started with a trial placement
	Client started an internship or perform voluntary work	10 (18%)
Intensive coaching (continuous process)	Client was supported for as long as they wanted you to support them	40 (70%)

		Number (%) of clients for whom this was executed (*N* = 41, i.e., number of clients who started any type of work/education)
Intensive coaching during employment (continuous process)	Client received coaching while employed	27 (66%), of which 20 received coaching from their IPS coach and 7 from someone else (e.g., an internal job coach)
	Client was coached when they wanted to start a new job when already in employment	10 (24%)

#### PWI

For a majority (62%) of clients who were assigned to PWI, the preparatory PWI was used. Table 2 shows the dose delivered for PWI. The complete PWI could theoretically have been conducted for 44 clients since they started (paid or voluntary) work or education. Of these 44 clients, for almost half (45%), the task analysis was completed by the client and job coach, and for 36% it was completed by the supervisor and job coach. It was often the case that later steps were carried out while previous steps were reported as not carried out. An example is that job coaches reported that for 39% of clients a brainstorm session had taken place, while the previous step of prioritizing and selecting obstacles had only taken place for 23% of clients. This seems contradictory, since for a brainstorm about solutions for obstacles, the obstacles need to be identified. It is likely that job coaches sometimes reported that they omitted certain steps of PWI because they were not carried out according to protocol.

**Table 2:** Dose delivered—participatory workplace intervention

Steps	Sub-steps	Number (%) of clients for whom this was executed (*N* = 58)
1. Preparatory PWI	Optional	36 (62%)

		Number (%) of clients for whom this was executed (*N* = 44)*
2. Task analysis & identification of obstacles *With client*	Task analysis and discussion about obstacles for maintenance of the job	20 (45%)
	Prioritize obstacles	14 (32%)
	Choose (maximum of 3) most important obstacles	18 (41%)
3. Task analysis & identification of obstacles *With supervisor*	Task analysis and discussion about obstacles for maintenance of the job	16 (36%)
	Prioritize obstacles	9 (20%)
	Choose (maximum of 3) most important obstacles	13 (30%)
4A. Brainstorm session	Discuss client and supervisor discrepancies in perceived tasks & obstacles	11 (25%)
	Choose a maximum of 3 mutual obstacles to work on	10 (23%)
	Brainstorm about possible solutions for each obstacle	17 (39%)
4B. Prioritize solutions	For each solution, assess feasibility and expected effectiveness	9 (20%)
	In consensus, choose the most fitting solutions	14 (32%)
4C. Action plan	Determine which steps should be taken to put solutions into practice	14 (32%)
	Describe who will do what, when, and how	15 (34%)
5. Evaluation	Evaluate whether solutions were carried out	12 (27%)
	Evaluate whether solutions took away or alleviated obstacles	12 (27%)
	Evaluate whether additional obstacles still exist	13 (30%)

*This is the number of clients for whom steps 1–4 could be executed (i.e., clients who started any type of work/education)

#### Intensity and Duration

The average number of minutes per week that job coaches spent on coaching their clients were similar for the IPS and PWI groups: 27 min of coaching per week for both groups (SD 14 for the IPS group, 16 for the PWI group). The average duration of coaching in the first 12 months was 46 weeks in both groups (SD 13 for IPS group and SD 14 for PWI group).

### Fidelity

#### IPS

The IPS fidelity review score was 92, which corresponds to “fair” (scores of 100 and higher are considered good, scores between 74 and 99 are considered fair, and scores below 74 are considered no IPS [21]). The external reviewers mentioned in their report that most IPS principles were applied. In contrast to the usual service of the municipality, whether clients were ‘ready to work’ or not was not taken into consideration if they received IPS. The collaboration within the municipality was mentioned in the report as a positive aspect (e.g., how job coaches could receive help from benefits counselors). Sufficient support regarding income and benefits counseling was offered, and all of the efforts that IPS coaches made were aimed at finding competitive employment. The main issue in the IPS fidelity was lack of integration between vocational rehabilitation and healthcare services. In the context of this study, it was not always possible for job coaches to contact healthcare providers and other caregivers: sometimes clients did not receive healthcare (while in some cases job coaches felt the client needed care), and sometimes clients did not give permission to contact their healthcare provider. Moreover, healthcare providers often did not feel comfortable talking about their patients due to privacy regulations. For young adults and adolescents, there was more often collaboration with healthcare professionals or family members than for adult clients, because there was more often a support network in place (consisting of e.g., parents, social workers, and educational professionals).

Finally, it was mentioned that job coaches had an average caseload of 38, which is larger than what is desirable for an IPS employment specialist (max. 20). It must be noted, however, that a maximum of 10 clients per job coach received IPS coaching. The remainder consisted of the regular caseload and thus of clients who did not participate in the study and received SAU.

#### PWI

We could not determine the fidelity of PWI due to a lack of information. Despite instructions to use the provided materials to conduct the intervention and to document the different steps and outcomes of PWI, only one of the eight job coaches in the PWI group used the intervention materials as intended. Most job coaches indicated that when carrying out PWI they used the materials barely or not at all. Therefore, we were not able to assess the general quality of the approach.

## Discussion

This study describes a process evaluation of IPS and PWI to increase labor market participation of people with work disabilities, conducted alongside an RCT. The main objective was to describe whether and to what extent IPS and PWI were executed according to protocol. The results showed that both interventions were challenging to carry out according to protocol in the municipal setting. For PWI the dose delivered was low: none of the intervention steps were carried out for more than 50% of clients. The fidelity of the PWI could not be assessed, since the intervention materials that were supposed to serve as documentation were barely used. The lack of use of intervention materials suggests a poor fidelity, however since in theory the intervention can still be carried out sufficiently without using the forms, the fidelity remains unclear. For IPS, dose delivered was reasonable: for almost all clients the job search indeed focused on competitive employment based on the clients’ preferences. However, contact with important members of the clients’ network (e.g., healthcare providers or family members) was realized for less than half of the clients. The lack of integration between employment services and healthcare services was also the main reason why the IPS fidelity measurement score was only fair.

This lack of integration is in line with other studies that have also mentioned similar challenges, even in the ‘traditional’ setting of IPS for people with mental illness [22, 23]. In that setting, the IPS coach is part of the treatment team of a mental health care organization, and thus collaboration between these professionals is facilitated by regular team meetings and working in the same building. If collaboration is already complicated in that setting, it is not surprising that it was even more challenging in the municipal setting of this study. Here, collaboration with other professionals was complicated because job coaches and healthcare professionals were working in different organizations, and clients did not always give permission to their job coaches to contact these healthcare providers. When clients did give permission, healthcare providers often seemed hesitant to talk about their clients with the job coach because they wanted to protect the privacy of their patient. Finally, it was often the case that clients did not receive any form of care, although job coaches sometimes felt that such care was needed. Unfortunately, job coaches can only advise their clients to apply for (health)care services, but they do not have the authority or right to directly refer someone to these services because they are social service providers and not health care professionals. Even though integration between healthcare providers and employment services seems challenging, it is important that efforts are made to increase this integration when IPS is carried out outside of the healthcare setting. Previous research has shown beneficial effects of integrated services compared to separated services [24, 25], and higher fidelity scores have been correlated to better employment outcomes [26, 27].

The low dosage of PWI we found is not in line with previous research: most studies on participatory workplace interventions found that the intervention was carried out for most clients [19, 28, 29]. One explanation for our results is that clients were unemployed at baseline and a workplace was a requirement for the main part of PWI; since 24% of clients did not start any work (or education), they could not receive the main part of the intervention. Previous studies on participatory workplace interventions were not dependent on finding a workplace, since they often included sick-listed workers [29] or involved other stakeholders (e.g., insurance physicians or labor experts) instead of an supervisor or employer [19, 28]. However, even when we only consider clients who started work or education, the dosage we found was low compared to these previous studies. In a study on a similar intervention by van Beurden et al., obstacles were identified, a brainstorm session took place, and an action plan was made for 53% of clients, whereas in our study the brainstorm session took place for 39% of clients and an action plan was made for only 34% of clients. It seems that especially the steps in which the client and supervisor both had to be involved were executed less often in our study. This might be because job coaches felt hesitant to initiate the intervention since it was a new and much more structured way of working than they were used to, or because they had to ask supervisors to put in quite a lot of time and effort.

### Strengths and Limitations

This study had several strengths. First, we used a framework that enabled us to conduct our process evaluation systematically and include multiple important process measures. To our knowledge, this is one of the first papers that investigated the execution of IPS outside of the mental healthcare setting, using a process evaluation that reports on the individual level and in such a systematic manner. Another strength is that we observed the implementation of the interventions for a long period of time (approximately 3-years, throughout the inclusion period and follow-up duration of the study) and thus gained more comprehensive information.

There are also limitations to this study. First, job coaches were assigned to an intervention based on their preference for practical reasons. It is possible that job coaches were thus very motivated to execute the intervention according to protocol. Therefore, implementing the interventions in daily practice, when it would be carried out by job coaches who did not volunteer for this way of working, is likely to be even more challenging. A second limitation is that we could not determine the fidelity of PWI because the method was poorly documented. Finally, certain restrictions regarding coaching at the workplace and face-to-face meetings due to COVID-19 measures influenced the way and the extent to which the interventions could be executed.

## Concluding Remarks

Both IPS and PWI showed to be challenging to implement in the daily practice of a large municipality in the Netherlands. The main barrier for IPS seemed to be a lack of integration of employment services with healthcare services. For PWI, absence of a workplace and the components in which multiple stakeholders had to be involved seemed to be challenges. Further investigation into specific barriers and facilitators is needed to improve implementation of both interventions. Additionally, because IPS is increasingly implemented in new settings and for new target populations, it is essential that more process evaluations are performed to identify how IPS is carried out and how contextual factors influence (work) outcomes.

## Supplementary Information

Below is the link to the electronic supplementary material.

Supplementary file1 (PDF 602 kb)

Supplementary file2 (PDF 695 kb)
