# Supplementary material for: Process Evaluation of Individual Placement and Support and Participatory Workplace Intervention to Increase the Sustainable Work Participation of People with Work Disabilities
Source: J Occup Rehabil. 2024 Jun 25;35(2):400–10. doi: 10.1007/s10926-024-10214-x (PMC12089157; doi:10.1007/s10926-024-10214-x)
Supplement: Supplementary file 1 — Supplementary file1 (PDF 602 kb) [file 10926_2024_10214_MOESM1_ESM.pdf]

Process Evaluation of Individual Placement and Support and Participatory Workplace Intervention to Increase the Sustainable Work Participation of People with Work Disabilities

E. Oude Geerdink[1], M.A. Huysmans[1], H. van Kempen[2], J.M. Maarleveld[1], J. van Weeghel[3], J.R. Anema [1]

[1]*Department of Public and Occupational Health, Amsterdam Public Health Research Institute, Amsterdam UMC, Vrije Universiteit Amsterdam, Amsterdam, 1081 BT, Netherlands*

[2]*Research and Statistics, City of Amsterdam, Amsterdam, The Netherlands*

[3]*Tranzo, Tilburg School of Social and Behavioral Sciences, Tilburg University, Tilburg, The Netherlands*

Appendix 1: Description of IPS

Table 3: The eight IPS principles [1] with the adaptations that were needed for implementation in the municipal setting

**IPS principle**	**Adaptations needed to the intervention and/or training?**
1) Focus on competitive employment	No adaptations were needed. However, even though job coaches were encouraged to search for jobs in regular settings (i.e., competitive work in a regular work environment, where clients work alongside people without work disabilities), they were also allowed to make use of their regular networks that included more sheltered workplaces, if these involved at least a regular employment contract and salary, and if this fitted the clients' wishes best.
2) Zero exclusion (every client who wants to work is eligible for services)	No adaptations were needed. However, in this study clients were expected to start a trajectory toward work, in contrast to the original IPS in which a trajectory starts when the client expresses a wish to work. For the study we aimed to include clients who were motivated to start a job and after inclusion in the study all clients could be assigned to IPS, regardless of their 'readiness to work.'

3) Integration of employment services with mental health treatment	Adaptations were needed because job coaches in the municipality did not work within a healthcare setting. Therefore, job coaches were instructed to list any healthcare providers, but also other formal or informal service providers (e.g., social workers, debt counsellors) and important family or friends. They then had to ask the client for permission to contact the most important people on this list. The aim of this contact was to achieve agreement on the goals of coaching toward work and sometimes also to gain advice from (health)care providers regarding what a client might need, or whether the job coach should consider important aspects such as symptoms or functional limitations, and how to take these into consideration during the job and job search.
4) Attention to clients' preferences: services align with clients' choices and IPS specialists help clients find jobs that fit their preferences and skills	No adaptations were needed.
5) Personalized benefits counselling	No adaptations were needed.
6) Rapid job search	No adaptations were needed. In the training, it was emphasized that this principle was also applicable to clients who in the usual service would have received pre-vocational training.
7) Targeted job development: IPS specialists build relationships with employers based on clients' interests	No adaptations were needed. Job coaches already had a comprehensive network and could use their existing work relations with employers and jobhunters, and were encouraged to keep expanding this network.
8) Time-unlimited and individualized support	No adaptations were needed

References

1. Bond GR, Drake RE, Becker DR. An update on Individual Placement and Support. World Psychiatry. 2020;19(3):390-1. doi: 10.1002/wps.20784.
