# Supplementary material for: Process Evaluation of Individual Placement and Support and Participatory Workplace Intervention to Increase the Sustainable Work Participation of People with Work Disabilities
Source: J Occup Rehabil. 2024 Jun 25;35(2):400–10. doi: 10.1007/s10926-024-10214-x (PMC12089157; doi:10.1007/s10926-024-10214-x)
Supplement: Supplementary file 2 — Supplementary file2 (PDF 695 kb) [file 10926_2024_10214_MOESM2_ESM.pdf]

Process Evaluation of Individual Placement and Support and Participatory Workplace Intervention to Increase the Sustainable Work Participation of People with Work Disabilities

E. Oude Geerdink[1], M.A. Huysmans[1], H. van Kempen[2], J.M. Maarleveld[1], J. van Weeghel[3], J.R. Anema [1]

[1]*Department of Public and Occupational Health, Amsterdam Public Health Research Institute, Amsterdam UMC, Vrije Universiteit Amsterdam, Amsterdam, 1081 BT, Netherlands*

[2]*Research and Statistics, City of Amsterdam, Amsterdam, The Netherlands*

[3]*Tranzo, Tilburg School of Social and Behavioral Sciences, Tilburg University, Tilburg, The Netherlands*

Appendix 2: Description of PWI

The process of PWI consists of three steps. In the first step, the tasks and the presence of possible corresponding obstacles are evaluated by the client and process leader and the supervisor and process leader, separately. In the second step, the process leader, supervisor, and client get together and reach consensus on the most important obstacles to address. A brainstorm session on solutions takes place and the most fitting solutions are put into an action plan. In the final step, an evaluation is conducted to determine whether the agreed upon actions have been carried out and whether the obstacles are solved. If important obstacles still exist, the process can start again at step 1 or 2. Actual PWI can only begin after a client started work, an internship, or education, but since professionals indicated they wanted to start coaching immediately, an optional introductory part was added to PWI: the preparatory PWI. This could be used during the search for competitive employment and aimed to identify and solve obstacles for starting employment. In the preparatory PWI, similar steps are conducted as in PWI, but with only the professional and the client as participants in the conversations.

Table 4 : Description of Participatory Workplace Intervention in steps

**Steps**	**Sub-steps**	**Who is involved**	**When does this take place**
0) Preparatory PWI	*Optional*	Process leader and client	While searching for competitive employment

1) Task analysis & identification of obstacles *with client*	Task analysis and discussion about obstacles for maintenance of the job	Process leader and client	Timing depends on when the client and supervisor both have had sufficient time to assess work functioning, content, and challenges of the job. This was generally between 2 and 6 weeks after starting employment
	Prioritize obstacles		
	Choose (maximum of 3) most important obstacles		
2) Task analysis & identification of obstacles *with supervisor*	Task analysis and discussion about obstacles for maintenance of the job	Process leader and supervisor	
	Prioritize obstacles		
	Choose (maximum of 3) most important obstacles		
3) A. Brainstorm session	Discuss discrepancies between the client and supervisor in perceived tasks and obstacles	Client and supervisor, guided by process leader	As soon as possible after the task analysis
	Choose a maximum of 3 mutual obstacles to work on		
	Brainstorm about possible solutions for each obstacle		
B. Prioritize solutions	For each solution, assess feasibility and expected effectiveness		
	In consensus, choose the most fitting solutions		
C. Action plan	Determine which steps should be taken to put solutions into practice		
	Describe who will do what, when, and how		
4) Evaluation	Evaluate whether solutions were carried out	Client and supervisor,	Timing depends on content of the action plan

	Evaluate whether solutions took away or alleviated obstacles	guided by job process leader	
	Evaluate whether additional obstacles still exist		
